# “We just get paid for 12 hours a day, but we work 24”: home health aide restrictions and work related stress

**DOI:** 10.1186/s12913-019-4664-2

**Published:** 2019-11-28

**Authors:** Jillian L. Shotwell, Eve Wool, Andrzej Kozikowski, Renee Pekmezaris, Jill Slaboda, Gregory Norman, Karin Rhodes, Kristofer Smith

**Affiliations:** 1 Northwell Health Solutions, Manhasset, NY USA; 2 Northwell Health Center for Innovations and Outcomes Research, Manhasset, NY USA; 3 West Health Institute, La Jolla, CA USA

**Keywords:** Managed long term care, Home based primary care, Formal caregivers, Home health aide

## Abstract

**Background:**

Home-bound patients in New York State requiring long-term care services have seen significant changes to their benefits due to turmoil in the Managed Long Term Care (MLTC) market. While there has been research conducted regarding the effect of MLTC challenges on beneficiaries, the impact of MLTC regulatory changes on home health aides has not been explored.

**Methods:**

Qualitative interviews were conducted with formal caregivers, defined as paid home health aides (HHAs) (*n* = 13) caring for patients in a home-based primary care program in the New York City metropolitan area. HHAs were asked about their satisfaction with the home based primary care program, their own job satisfaction, and whether HHA restrictions affect their work in any way. Interviews were audio-recorded, transcribed, and analyzed.

**Results:**

Two main themes emerged: (1) Pay, benefits and hours worked and (2) Concerns about patient well-being afterhours**.** HHAs are working more hours than they are compensated for, experience wage stagnation and loss of benefits, and experience stress related to leaving frail clients alone after their shifts end.

**Conclusions:**

HHAs experience significant job-related stress when caring for frail elderly patients at home, which may have implications for both patient care and HHA turnover. As government bodies contemplate new policy directions for long-term care programs which rely on HHAs the impact of these changes on this vulnerable workforce must be considered.

## Introduction

Home Health Aides (HHAs) and formal caregivers are essential to many with long-term care needs, particularly homebound, frail elderly patients. Many work for Managed Long Term Care (MLTC) organizations and some are paid directly by the patient or their family. Their role is to provide assistance with patient activities of daily living, and support for managing chronic conditions. Depending on their employer, HHAs are subject to a variety of state-level regulations that determine the pay they receive, the number of hours they are eligible to work, and their scope of work [1, 2].

Changes in HHA regulations have the potential to create misalignment between the intention of these formal caregivers and their ability to provide quality care to the sickest of patients. Job stress and turnover in various healthcare settings has proven to have significant negative impact on patients that depend on these services for quality care [3, 4]. The objective of this study is to explore the impact of state regulations, such as the MLTC program regulations in New York State, on HHA’s perceived ability to provide patient care.

State policy, such as New York State’s 13-h rule [5], can constrain the ability of patients to receive the care that they need, and negatively impacts the workplace environment for the formal caregiver. The 13-h policy allows MLTC companies to mandate 24 h shifts, while only paying for 13 h of work. While federal funding is allocated for providing the services to eligible Medicare and Medicaid beneficiaries, home care services are provisioned using different financial models at the state level. Since 2016, New York State MLTC programs have made headlines, with plans to reduce or eliminate coverage for thousands of beneficiaries in the New York City area. MLTC programs provide services to chronically ill and disabled people in their homes; with mandatory coverage for those who are dually-eligible for Medicare and Medicaid. Among the many services that MLTCs provide is the provision of HHAs to qualifying beneficiaries. Over the past 2 years, several major MLTCs have shut down their programs in the New York Metropolitan area, citing mounting financial losses due to premiums which do not support the costs of the mandated services [6, 7].Specifically, MLTC company executives have stated that Medicaid’s premiums for severely ill patients is not adequate for the high-needs of these members [6]. The sudden closing of a number of large MLTCs has resulted in beneficiaries quickly enrolling in the remaining MLTC plans. State policy prevents patients from losing their MLTC benefits due to market instability; beneficiaries are automatically assigned to a new MLTC if their plan closes, and they are entitled, at least initially, to care continuity around both providers and intensity of services [8].

While there has been documented concern regarding the effect of regulatory challenges on beneficiaries, the impact that changes on the regulatory status on HHAs and their work has not been explored. HHAs employed in New York State work long, unpredictable hours with physically strenuous and mentally draining tasks, such as housework, preparing patients for appointments, and moving bedbound patients, and are not paid for all the hours that they work [9]. In 2014, HHAs settled a class action lawsuit addressing “the 13-hour policy” which allowed MLTC plans to mandate 24 h shifts, while only paying for 13 h [10]. MLTCs have resolutely opposed changing the 13-h policy, stating that paying for the full 24 h could bankrupt an already struggling industry. HHA funding and financial challenges are occurring country-wide, with 26 states rolling back mandatory enrollment for dually-eligible beneficiaries in managed long term care programs [11].

The objective of this study is to explore the impact of MLTC restrictions and changes on HHAs and their perceived ability to provide patient care. Using semi-structured qualitative interviews, this study explores themes of job satisfaction, patient safety, employee remuneration, and regulatory uncertainty. The findings will help to identify the consequences of governmental changes to this fragile system.

## Materials and methods

Semi-structured interviews were conducted with formal caregivers (*n* = 13, Table 1) of home-bound chronically ill patients participating in a home-based primary care program in the New York City Metropolitan area of Nassau, Suffolk, and Queens Counties (Additional file 1). Interviewees were either certified HHAs (*n* = 9), through a MLTC or the CDPAP (Consumer Directed Personal Assistance Program) (*n* = 1), or were self-employed, private caregivers (*n* = 3). The interviews analyzed in this study were conducted as part of a larger study designed to better understand the strengths and areas for improvement of a home-based primary care practice from the perspective of those involved in the delivery and receipt of care. The setting of our interviews was in patient homes being served by Northwell Health House Calls program. Formal caregivers were interviewed privately in a room separated from their clients and the client’s family whenever possible. Two participants were interviewed in the same room as their client, as the client had advanced dementia and could not be left alone. Researchers recruited a purposive sample of cognitively intact patients, family caregivers, and formal caregivers to explore program satisfaction and gather opinions on the possible incorporation of telehealth into the House Calls practice.

**Table 1:** Demographics of Formal Caregivers Interviewed

Employment Type	N Interviewed	Gender	Length of time working with client
Agency caregiver (MLTC funded)	9	Females *n* = 8 Males *n* = 1	2 months- 16 years
CDPAP employee	1	Females *n* = 1	2 years
Private paid caregiver	3	Females *n* = 2 Males *n* = 1	3–4 years

Interview guides were developed by qualitative researchers (RP, KVR, AK) and reviewed by the research team. The interviews were initially designed to solicit caregiver views regarding utilizing telehealth monitoring in the home. However, when discussing barriers to care, a number of interviewees spontaneously expressed their concerns surrounding work stress. The purpose of the current paper is to specifically focus on caregiver responses related to state-level restrictions and working conditions. Once this issue was raised in early interviews, subsequent caregivers were asked about whether policies or restrictions limited their ability to care for their client. Formal caregivers were specifically probed regarding home health agency restrictions, and whether their job as a formal caregiver has been affected by these regulations.

Northwell Health’s House Calls program is a home-based primary care program serving homebound and frail patients living in Nassau, Suffolk, and Queens Counties in New York. The HHAs are arranged separately by the family, but work with the House Calls’ staff to coordinate care. Formal caregivers were referred to the study team by House Calls staff, and then family members of patients were contacted to inquire if interviews could be conducted in the patient’s home. After receiving patient or family approval, formal caregivers were told the purpose of the study and asked if they would like to participate in a confidential interview, stressing the voluntary nature of their participation. Consenting HHAs signed informed consent prior to being interviewed or recorded. Research staff obtained written consent and conducted the interviews at a scheduled appointment during working hours in the patient’s home. The study was approved by the Northwell Health Institutional Review board as an exempt quality improvement initiative.

Interviews were audio recorded and professionally transcribed by an independent transcription service. Twelve interviews were completed with 13 formal caregivers; the interview length ranged between 7 and 23 min with a mean of 16 min. Two formal caregivers were interviewed together. The transcripts were subsequently entered into NVIVO 11 [12], a qualitative data software. Data were analyzed using thematic analysis and coded by 3 qualitative reviewers (JLS, EEW, AK). Reviewers met together after an initial read of all transcripts to develop theme nodes for thematic coding. JLS and EEW each coded all interviews, with AK coding acting as a third reviewer on two of the interviews.

## Results

After analysis of the interview transcriptions, two major themes emerged regarding how restrictions on HHAs affected the formal caregivers’ ability to work: (1) Pay, benefits and hours worked and “after hours” work expectations. During the interviews formal caregivers frequently referenced stress regarding the impact of changing hours and decreasing pay (*n* = 4). (2) Interviewees also expressed a feeling that their clients required more help than their hours allotted them, leaving the formal caregiver feeling torn between caring without pay and leaving the patient alone (*n* = 5). These themes emerged specifically from caregivers who worked currently as MLTC-funded Home Health Aides (*n* = 9) or previously worked as an MLTC funded Home Health Aide (*n* = 1).

### Theme 1: pay, benefits, and hours worked

Formal caregivers of home-bound patients expressed work-related stress induced by decreases in pay, benefits, and hours worked per week. Without specific inquiry regarding pay and benefits, several caregivers discussed their pay, and that they were being paid for fewer hours than they were scheduled to work per week. One Certified HHA expressed frustration that while they worked 24 h per day, they only get paid for 12 of those hours, and received no benefits. “*… we just get paid for 12 hours a day. But we work … 24 hours … We fight for it and we have no more holidays, no more vacation, no more sick day [s].”* Another stated that they worked 72 h per week, but were only paid for 36, or half of their hours worked. “ “*… 12 by 3 – 36 hours … but it’s 24 hours live-in. That’s how they call it. But we do more – 24 … , three days a week.”* One caregiver, in addition to discussing similar payment parity, shared their hourly wages. Based on their description, their hourly pay averages approximately $5.17. “… *Our salary is $10.35 in 12 hours. But we have 24 hours. It’s not fair*.”

HHAs also discussed challenges regarding the predictability of their wages. Specifically, an HHA stated that the company they worked for was lowering their salary over time, and changing the salary frequently. “*Little by little, they do something that makes our salary low. And now, I don’t know what’s again. Every month, it changes*.” Another expressed concern that their hours worked per week would be cut. *“That’s a problem now. They[‘re] gonna cut the hours. They don’t want people to work more than 40 hours … I work … 8:00 to 8:00. So they’re gonna cut all those hours … And then they say we can’t work more than 40 hours. That’s what they say.”* Two caregivers interviewed together had been in their position for years, but had not received any increase to their pay rate. *“That’s why we stay here very long. But right now, we are – we need some to increase our salary because we are working almost seven years now”.* It was apparent from these interviews that threats to cut pay, stagnant incomes, and unpredictable or long hours were a stressor for caregivers.

### Theme 2: concerns about patient well-being after hours

Formal caregivers noted they had a dilemma between caring for their homebound clients adequately and complying with scheduled hours of care per week. Responses regarding care hours came from both those who were working as agency HHAs and from a private caregiver who had previously worked as an agency HHA. Several caregivers stressed they often stayed past their paid hours due to the needs of their frail client. “*… every time we have work... We do the best we can do. And for her … especially she needs us. That’s why we cannot go home. That’s the best we can do really. We stay here as long as lady’s there.”* Another caregiver stayed past their care hours regularly due to the demands of the job “*So I’m here late, after 8:00, because something pop up, you know?* If a client is frail and needs assistance to an appointment that runs long, caregivers may be caught in a dilemma on whether to leave the patient or assist them to get back home without being paid for their time. *“Well, sometimes … if you go out to the clinic and the time has passed that you[‘re] supposed to get home, it’s kind of unfair for you to leave the patient … but you have to leave a certain time.”*

One caregiver, who chose to work privately because of his experience as an HHA, said he switched agencies because of hours restrictions. *“In my experience, what limited me was just agency policies and rules … And there’s times where if – or certain hour – hours isn’t given to the patient, you’re not allowed to go past that hour. And I’ve actually quit quite a few jobs because of that.”* The same caregiver stayed late at his client’s house out of empathy for the distressed family caregiver, who was left alone to care for the patient after work hours. *“Sometimes she’s here with him and she has to work too and she’ll come home. She can’t even go back to work, no sleep. If I’m here, I’m most likely never going to leave her in that condition-to just be up an entire week, which she has done … So I’m not going to leave. That’s just who I am.”* Surprisingly, one interviewee even paid a friend to work during their off-hours to provide care when they could not. *“I tell you the truth. Sometimes, I get my friend. I pay for – just use my name and just take care of her, just not to leave her.”*

## Discussion

By 2030, it is expected that 20% of the population will be aged 65 or older and these individuals will likely require additional long-term care services [13]. These demographic trends have already led to growth in the HHA workforce, doubling from 2007 to 2017 [14, 15]. Turnover of health care workers is expensive, and negatively impacts both the quality of care for patients and staff- patient relationships [16–18] Our results describe factors that contribute significantly to HHA job dissatisfaction, and eventually, job turnover. These factors may have larger implications for both quality of patient care and patient quality of life.

The analysis of the interviews revealed financial and regulatory factors that cause significant stress for formal caregivers when assisting frail, homebound clients. Concerns about decreased pay and benefits and leaving clients alone after working hours were specific to the five caregivers currently working for MLTC programs, and the caregiver who had worked as a formal agency caregiver in the past. Responses regarding decreased pay and benefits were expressed without explicit inquiry into these topics; participants were asked only about the general impact of restrictions on caregiving.

MLTC plans in New York State are in significant financial crisis with 61% of New York State MLTCs operating at a loss in 2015 due to state underfunding [19]. Home care policy makers have warned that sustained MLTC losses will eventually impact patient access to care, the quality of services, and the workforce providing services [19, 20]. The range of hours that New York State HHAs are funded to work by Medicaid is significantly higher than that of other states, suggesting that the challenges of funding hours might be even greater across the country. Our findings suggest that chronic financial strain on MLTC programs may already be affecting formal caregivers who provide care for MLTC beneficiaries. Formal caregivers cited increased work-related stress from decreased compensation, benefit loss, and concerns that homebound patients required more care than their allotted hours. The later concern may reflect the growing push by remaining MLTC programs to cut financial losses through reductions in hours provided to members.

While prior studies have suggested the importance of alleviating job-related physical burden of working as a home health aide [21], our study identifies the need for policy changes that also address financial and emotional stress that is experienced by HHAs. It is important for the healthcare community to understand job stress in the HHA population, as it contributes to turnover, burnout, and ultimately, quality of care for homebound patients. Efforts to investigate the financial and emotional as well as the physical burdens placed on HHAs by changes in health care policy and financing are increasingly important, given the increasing need for quality long-term care on the national level. In qualitative interviews, HHAs in California reported efforts to increase their client loads to earn an income sufficient to pay their rent and monthly expenses [22]. These HHAs attributed the undervalued nature of their work as well as their lack of leverage as the reason behind insufficient monetary compensation. Our findings support work in other states where HHAs report similar themes regarding loyalty to their clients that can result in pressure or concern causing them to work overtime without being paid [23]. Our findings and the existing literature suggests that themes of work-related stress are shared by HHAs throughout the United States and policy changes which exacerbate such pressures should be reconsidered.

This study has several limitations that are common to qualitative studies. The formal caregivers interviewed were identified as willing by the Home Based primary care program’s staff, creating the potential for selection bias. We used convenience sampling for a number of interviews. For example, if an interview with a family caregiver or patient was scheduled, we would often interview the formal caregiver if they were available. Therefore the formal caregivers who were interviewed may not be representative of the views within a larger population of HHA workers. Importantly, questions about wages and hours were not part of the interview guide but were raised independently by the majority of the HHAs who were interviewed. It is notable that the self-employed, private caregivers were less likely to mention challenges to wages or hours worked, suggesting that those working for MLTC agencies are most impacted by the themes raised in this paper. However, given that those interviewed were all working in one program in the New York metropolitan geographic area, it is important to state that results presented here are hypothesis-generating and should be used to inform larger investigations in other geographic areas.

## Conclusion

The formal caregivers in this qualitative study discussed several challenges regarding caring for homebound elderly patients. These challenges have only accelerated with the changes and restrictions imposed by New York State’s struggling MLTC programs. Several caregivers noted working more hours than they were compensated for, both as a part of their expected weekly work schedule and due to a perception that their client could not safely be left alone. In the MLTC market, it is important to understand the impact of state regulations on formal caregivers and to make sure that as state regulations are implemented to stabilize the program, the needs of formal caregivers are strongly considered. Community-dwelling patients with chronic disease burden will suffer substantially if the caregiver workforce is further stressed.

## Supplementary information

**Additional file 1.** Caregiver Interview Guide, Interview guide specifying questions asked to caregivers during semi-structured interviews.

## Data Availability

Data sharing is not applicable to this article, as no datasets were generated or analyzed during the current study. Transcriptions of interviews with participants may not be shared, as information contained in these transcriptions may identify participants.

## Abbreviations

CDPAP: Consumer Directed Personal Assistance Program
HHA: Home Health Aide
MLTC: Managed Long Term Care
