# Supplementary material for: “We just get paid for 12 hours a day, but we work 24”: home health aide restrictions and work related stress
Source: BMC Health Serv Res. 2019 Nov 28;19:907. doi: 10.1186/s12913-019-4664-2 (PMC6883531; doi:10.1186/s12913-019-4664-2)
Supplement: Supplementary file 1 — Additional file 1. Caregiver Interview Guide, Interview guide specifying questions asked to caregivers during semi-structured interviews. [file 12913_2019_4664_MOESM1_ESM.docx]

Formal Caregiver Semi-Structured Interview Guide

Participant’s ID: __________

Status of Patient Caregiver is caring for:  Chronic  Complex  Advanced

Number of hours per week caring for patient: ____ hours

Length of time working with patient (in months): _____ months

Formal (through agency) or informal (off-the-books) caregiver: _______________

Primary Care Provider for Patient:  Physician  Nurse Practitioner

Current Visit Schedule: ________________________________

Date of Interview: __________

Initials of Interviewer: _______

[SAY] Thank you so much for allowing me to interview you for our study of the Northwell House Calls program. Before we begin, I just want to give you a bit of background on why we are doing these interviews.

This home-based health program, called House Calls, serves over 1,000 patients in the Nassau, Suffolk, and Queens County area. Doctors, nurses, and social workers travel to patients’ homes to give primary care services and support to patients who have a hard time leaving the house. Because the program has been very successful, we hope to grow it so that we can help even more patients that need it in this area. From this interview, I want to learn more from you about the strengths and areas to improve in the program, as well as your opinion on how we can both grow the program and keep patients home and healthy. We want to make sure that so that patients, families, caregivers, and program staff remain happy being a part of House Calls.

Do you have any questions about this before we begin?

1. Overall, what was your experience with the *House Calls* program?
   1. Probe: What about *House Calls* do you find most beneficial/meaningful/comforting?
   2. Probe: What about *House Calls* do you like most? What are its strengths?
   3. Probe: What about *House Calls* do you not like? What are its weaknesses?
   4. Probe: What would you change to make *House Calls* better?
   5. Probe: Did you experience any barriers to fully benefitting from *House Calls? What were the barriers?*
   6. Probe: How do you feel about the number of times *House Calls* staff visited the patient’s home? Should it remain the same/increase/decrease? Why? What would be the optimal number of visits? Why?
   7. Probe: How do you feel about interacting with *House Calls* staff? What was particularly beneficial/meaningful/comforting? What was not so beneficial/meaningful/comforting?
2. What, if anything, do you feel limits your ability to care for the patient?
   1. Probe: Do you feel regulations (e.g. not being allowed to take vital signs or give medications) limits your ability to care for the patient? Why or why not?
   2. Probe: Do you feel that the training you received so far limits your ability to care for the patient? Why or why not?

*Now let’s talk about changing the House Calls program by adding technology. This would consist of you using a computer based communication tool to share vital signs data like weight, blood pressure, heart rate etc. to let us know if the patient needs additional help.*

1. How would you feel if we added home health technology to increase communication between you and the *House Calls* team as part of the patient’s care?
   1. Probe: How would you feel about getting free training and credentialing for a health technology to monitor your patient’s health?
   2. Probe: Would adding this technology make you feel comfortable/uncomfortable? Why?
   3. Probe: Would it be helpful/unhelpful? Why?
   4. Probe: Would there be any barriers for you to use this technology? What are they? How comfortable or uncomfortable are you with technology?
   5. Probe: How would you feel about using health technology to communicate with the care team?
   6. Probe: Would the health technology make caring for the patient easier/more difficult? Why?
   7. Probe: Would you feel you have more/less support with this technology? Why?
2. If technology allowed us to see how the patient is doing remotely, how would you feel if we substituted some of the live visits with video visits?
3. How would you feel if the primary team leader is a Nurse Practitioner instead of a Physician (*interviewer:* *show diagrams* to *patients*)?
4. Do you have any other recommendations about the *House Calls* program?
5. Do you have any questions/comments about what we had talked about today?

| **Comments: INTERVIEWER,** use this space to summarize how the interview went, including the mood, facial expressions and body language of the participant during the interview. |
| --- |
